# Epidemiological trends in autism and other neurodevelopmental disorders in Kazakhstan (2016–2022): a regional and national perspective

**DOI:** 10.3389/fpsyt.2025.1520460

**Published:** 2025-06-03

**Authors:** Karina Nukeshtayeva, Zhanerke Bolatova, Aza Galayeva, Nurzhamal Shintayeva, Gulmira Zhanalina

**Affiliations:** School of Public Health, Karaganda Medical University, Karaganda, Kazakhstan

**Keywords:** autism, Kazakhstan, trend, developmental disorder, epidemiology

## Abstract

**Background:**

This study investigates the epidemiological trends of autism spectrum disorder and other neurodevelopmental disorders in Kazakhstan from 2016 to 2022.

**Methods:**

Utilizing national healthcare databases, we analyzed the incidence and prevalence of childhood autism, atypical autism, and neurodevelopmental disorders across various regions of Kazakhstan.

**Results:**

Our findings reveal a nearly fivefold increase in the diagnosis of childhood autism and a fourfold increase in atypical autism over the seven-year period, with both trends showing statistical significance (p < 0.01). However, no significant trend was observed for broader neurodevelopmental disorders. Regional disparities were evident, with northern regions showing higher rates of ASD diagnoses compared to the southern regions, likely influenced by factors such as healthcare infrastructure, urbanization, and environmental pollution. Astana showed a marked increase in childhood autism prevalence, reaching 263.7 per 100,000 children by 2022.

**Conclusion:**

Data highlight an important public health trend in Kazakhstan, where the primary incidence of autism spectrum disorders is on the rise, reflecting improved recognition and diagnostic practices.

## 1 Introduction

Child health and development are fundamental components of public health, influencing not only individual well-being but also the prosperity and stability of entire communities and nations (1). Prioritizing early childhood development within public health strategies is crucial for fostering a healthier, more equitable, and sustainable future (2).

Childhood mental and developmental disorders, which include cognitive, emotional, and behavioral disorders, have significant and widespread adverse effects on psychological and social well-being. Establishing a global epidemiology of mental disorders is challenging due to substantial data gaps across many regions and the influence of cultural differences on presentation and diagnosis (3, 4). For example, the reported prevalence of childhood developmental disorders varies widely across studies, ranging from 3% to 30%, primarily due to differences in data collection methods, diagnostic criteria, and assessment instruments (5).

Autism spectrum disorder (ASD) is a developmental disorder that impacts social interaction, communication, learning, and behavior. According to the International Classification of Diseases, 10th Revision (ICD-10), which is currently used by child psychiatrists in Kazakhstan for diagnostic purposes, autism falls under the category of Pervasive Developmental Disorders, which includes eight diagnoses such as Childhood Autism, Atypical Autism, and Asperger’s Syndrome. Both ICD-10 and DSM-IV define autism as a behaviorally determined syndrome characterized by impairments in social interaction and communication, as well as repetitive routines and restricted interests (6). Childhood Autism is defined by impaired development before age three and abnormalities in social interaction, communication, and repetitive behaviors. Atypical Autism differs by having a later onset or incomplete manifestation of these core areas, often presenting milder symptoms (7, 8).

Asperger’s Syndrome, distinct from other autism diagnoses, is characterized by the absence of general delay in language or cognitive development. However, it is often associated with pronounced motor clumsiness and a strong tendency for persistent behavioral abnormalities into adolescence and adulthood. Psychotic episodes may occasionally develop in early adulthood (9).

DSM-5 replaced the subtypes from DSM-IV (e.g., Asperger’s, PDD-NOS) with a single diagnosis—autism spectrum disorder —to improve diagnostic consistency and reflect the spectrum nature of autism. It also removed Rett Syndrome and Childhood Disintegrative Disorder from the ASD category and combined social and communication deficits into one domain (10, 11).

Similarly, ICD-11 adopted a more unified approach by merging the autism diagnoses from ICD-10 into a single diagnosis: autism spectrum disorder. This revised framework acknowledges autism as a dimensional condition that varies in presentation and severity rather than consisting of distinct categories. ICD-11 moved away from rigid diagnostic criteria and introduced specifiers to capture variations in intellectual and language abilities, presence of developmental regression, comorbid psychiatric or medical conditions, and known genetic or environmental etiologies (12). Notably, unlike ICD-10, which required symptom onset before the age of three, ICD-11 implements a more flexible developmental onset criterion, allowing for later symptom emergence or retrospective recognition of early signs (13).

Although ASD can be diagnosed at any age, it is classified as a “developmental disorder” because symptoms typically manifest within the first two years of life (14). ASD encompasses a range of diagnoses that were previously categorized under “Pervasive Developmental Disorders” in the 10th revision of the International Classification of Diseases.

Early studies on the prevalence of ASD, conducted up to the year 2000, estimated a prevalence of 10 per 10,000 individuals (15). However, a recent systematic review suggests that approximately 1 in 100 children worldwide are diagnosed with ASD (16).

In Kazakhstan, ASD is classified as a mental and behavioral disorder and is often accompanied by other developmental disorders and mental illnesses (17, 18). This combination of specific deficits and high comorbidity makes ASD one of the most disabling developmental disorders, imposing a significant economic burden (19–22). Research on the prevalence of autism began in the 1960s and 1970s, predating the formal inclusion of ASD in international diagnostic classifications and the establishment of standardized diagnostic criteria. Initial studies from this period estimated the prevalence of ASD to be between 0.5 and 0.7 cases per 10,000 individuals. Although these early estimates were based on limited data and varied methodologies, they provided the foundation for understanding the disorder.

Since the 1970s, research on ASD prevalence has expanded significantly, encompassing multiple regions and at least 37 countries (23). Despite this progress, prevalence data remain insufficient in many low- and middle-income countries, underscoring a critical gap in global health research. Additionally, studies on ASD prevalence in underrepresented areas often have small sample sizes and sometimes omit diagnostic confirmation (24–26). Notably, there has been a significant increase in the reported prevalence of ASD since the late 1990s, which can be attributed to improved access to diagnostic and intervention services, increased awareness of ASD among professionals and the public, and a greater recognition that ASD may co-occur with other developmental disorders (27).

Kazakhstan, the world’s largest landlocked country, is home to approximately 19 million people. It is ethnically diverse, with Kazakhs comprising around 68% of the population, followed by Russians (nearly 20%), and smaller groups such as Uzbeks, Ukrainians, and Uyghurs. The country’s cultural landscape reflects a complex blend of Turkic, Islamic, and post-Soviet influences, which significantly shape societal perceptions of health, disability, and social inclusion. These cultural frameworks deeply influence how autism is viewed and addressed.

During the Soviet era, disability was often regarded as a deviation from the ideal of “normalcy,” to be treated or hidden from official records. Statements such as “There are no disabled people in the USSR” were not uncommon (28, 29). This reflects the widespread stigmatization and marginalization of individuals with physical and mental impairments throughout the former Soviet Union (30). Even after independence, persons with disabilities in many post-Soviet states, including Kazakhstan, continue to be described as an “invisible group” (31).

The Soviet legacy contributed to a persistent culture of stigma surrounding mental health, hindering open discourse and acknowledgment of conditions such as ASD. As a result, many families have avoided seeking diagnosis or support due to fear of social ostracism. This historical backdrop has had lasting effects on the recognition and reporting of autism in Kazakhstan.

## 2 Methods

### 2.1 Variables and primary data collection

This study is a retrospective analysis of secondary data. The primary variables analyzed were:

Primary Incidence of Neurodevelopmental disorders: The annual number of newly diagnosed cases of Neurodevelopmental disorders per year per 100,000 population aged 0–17 years, based on data from mental health centers in Kazakhstan.

Prevalence of Neurodevelopmental disorders: The total number of children diagnosed with Neurodevelopmental disorders per 100,000 population aged 0–17 years, according to data from Kazakhstan’s mental health centers.

Primary Incidence of Childhood Autism: The annual number of newly diagnosed cases of Childhood Autism per 100,000 population aged 0–17 years, as reported by mental health centers in Kazakhstan.

Primary Incidence of Atypical Autism: The annual number of newly diagnosed cases of Atypical Autism per 100,000 population aged 0–17 years, based on data from mental health centers in Kazakhstan.

Prevalence of Childhood Autism: The total number of children diagnosed with Childhood Autism per 100,000 population aged 0–17 years, according to data from Kazakhstan’s mental health centers.

Prevalence of Atypical Autism: The total number of children diagnosed with Atypical Autism per 100,000 population aged 0–17 years, as reported by the mental health centers in Kazakhstan.

The annual statistical report “Mental Health Service,” compiled by the Republican Scientific and Practical Center for Mental Health, was utilized to obtain data on the incidence and prevalence of Autism in Kazakhstan from 2016 to 2022.

In cases where autism is suspected, primary healthcare physicians in Kazakhstan initiate a referral to the regional mental health center for specialized diagnostic evaluation. A child psychiatrist at the regional mental health center conducts diagnostic procedures in accordance with national clinical protocols (32). The clinical protocol for the diagnosis and treatment of autism spectrum disorders in Kazakhstan is based exclusively on the ICD-10. Upon identification of an ASD case, the child psychiatrist checks the electronic information system for mental and behavioral disorders to determine whether the child is already registered. If the diagnosis is established for the first time, the psychiatrist is required to enter the information into the system within three days, assigning the child to the statistical registry. In cases where the diagnosis had been established previously but is not present in the database, the record is added accordingly. If the information is already present, it is supplemented as necessary. Data on newly diagnosed cases and all registered children with autism across territorial mental health centers are collected and aggregated by the Republican Scientific and Practical Center for Mental Health. These data are made publicly available through annual statistical reports (33).

### 2.2 Statistical analysis

We employed linear regression analysis to identify statistically significant trends in the Primary Incidence of Neurodevelopmental disorders, Childhood Autism, and Atypical Autism in Kazakhstan during the period from 2016 to 2022. Statistical analysis was conducted using R-Studio software (version 1.2.5033, Posit, PBC, Vienna, Austria). A two-sided p-value of <0.01 was considered statistically significant.

## 3 Results

### 3.1 Primary incidence of neurodevelopmental disorders, including childhood and atypical autism, in the pediatric population from 2016 to 2022

**Figure 1:**
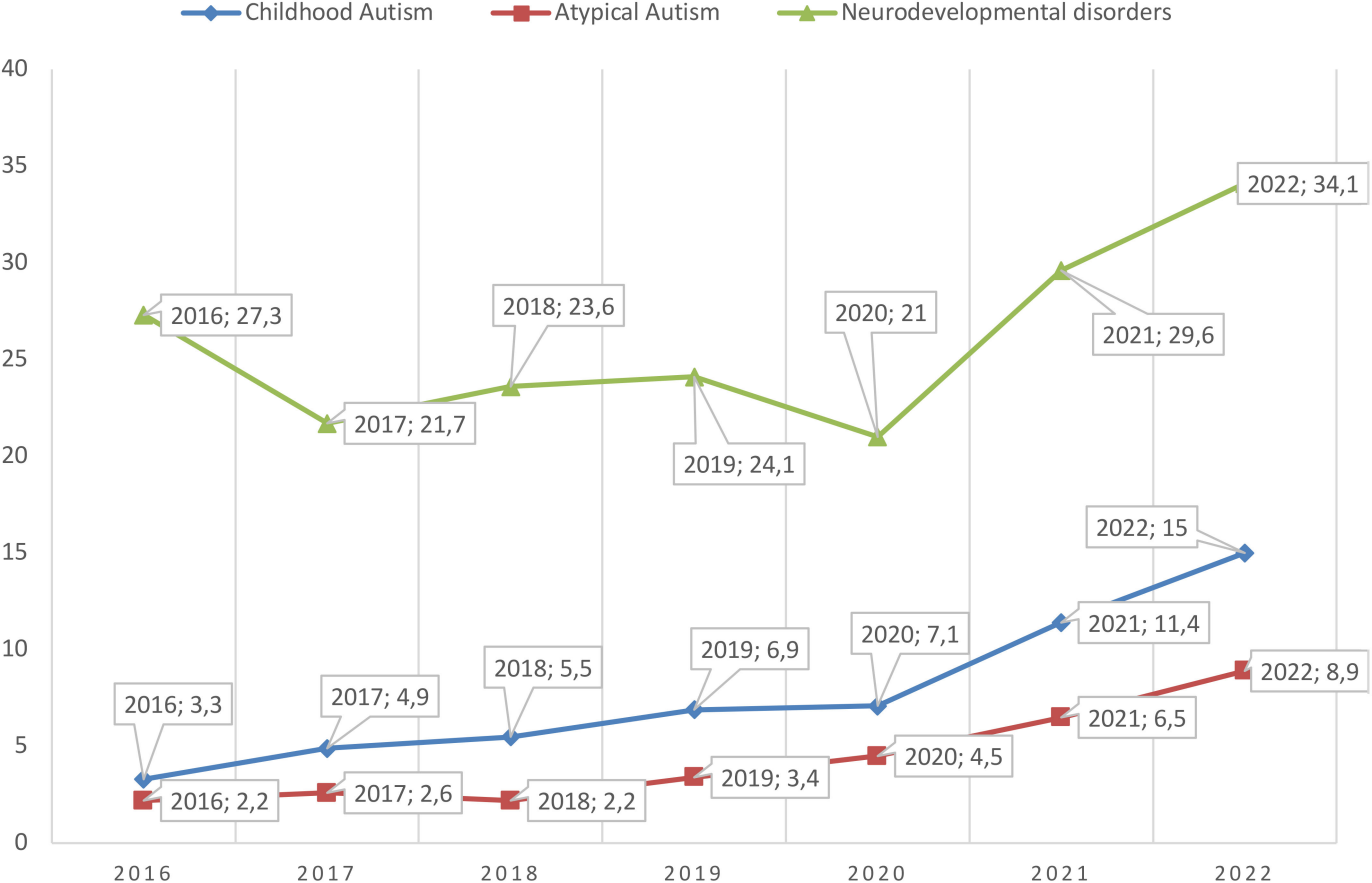
Primary incidence of neurodevelopmental disorders, childhood autism, and atypical autism per 100,000 children aged 0–17 years in Kazakhstan for 2016-2022.

The primary incidence of diagnoses of Childhood Autism and Atypical Autism increased significantly over the 7-year period. As illustrated in **Figure 1**, the national trend for newly diagnosed cases of Childhood Autism showed an almost fivefold increase, while the primary incidence of Atypical Autism rose fourfold. Additionally, the primary incidence of Neurodevelopmental disorders among children increased from 27.3 per 100,000 in 2016 to 34.1 per 100,000 in 2022.

**Table 1** clearly indicates that the increase in the primary incidence of both Childhood Autism and Atypical Autism is statistically significant. The data suggest that the primary incidence of Childhood Autism among children in Kazakhstan increases by an average of 1.78 cases per year, while the incidence of Atypical Autism increases by an average of 1.08 cases per year (p = 0.002, CI=1.03; 2.52 and p = 0.003, CI=0.55; 1.61 respectively). In contrast, the increase in newly registered diagnoses of Neurodevelopmental disorders overall is not statistically significant (p > 0.05, CI=-0.89; 3.29).

**Table 1:** Linear trend analysis coefficients of primary incidence of neurodevelopmental disorders, childhood and atypical autism in Kazakhstan from 2016 to 2022.

Dependent variable	*b*	*P*-value	R^2^
Neurodevelopmental disorders	1,20	0,20	0,16
Childhood Autism	1,78	<0,01	0,85
Atypical Autism	1,08	<0,01	0,81

**Table 2** presents the coefficients from the linear trend analysis models for the regions of Kazakhstan over the period 2016-2022. A negative coefficient, indicating a decreasing trend in the primary incidence of neurodevelopmental disorders, was observed in the Atyrau, Karaganda, South Kazakhstan, and North Kazakhstan regions. However, none of these declines were statistically significant, with p-values greater than 0.05. In contrast, an increasing trend was observed in all other regions, including the cities of Almaty and Astana. Notably, this increase was statistically significant in the Aktobe, Almaty, West Kazakhstan, Zhambyl, and Kyzylorda regions, as well as in the cities of Almaty and Astana (p < 0.05).

**Table 2:** Coefficients of trend analysis for the primary incidence of neurodevelopmental disorders, childhood autism, and atypical autism in children by regions of Kazakhstan from 2016 to 2022.

Region of Kazakhstan	Neurodevelopmental disorders			Childhood Autism			Atypical Autism		
	*b*	p	R^2^	*b*	p	R^2^	*b*	p	R^2^
Akmola	2,17	>0,05	0,02	4,63	**<0,05**	0,63	0,65	>0,05	0,14
Aktobe	3,75	**<0,001**	0,95	2,65	**<0,001**	0,89	0,17	>0,05	0,13
Almaty	1,89	**<0,05**	0,62	0,74	**<0,01**	0,73	0,17	**<0.05**	0,69
Atyrau	-0,34	>0,05	0,19	0,89	>0.05	0,27	1,67	**<0,05**	0,69
West Kazakhstan	3,63	**<0,01**	0,73	2,82	**<0,05**	0,62	0,6	**<0,05**	0,61
Zhambyl	3,59	**<0,001**	0,89	2,39	**<0,05**	0,49	0,42	**<0,05**	0,58
Karaganda	-3,12	>0,05	0,09	1,42	**<0,05**	0,49	0,95	**<0,05**	0,46
Kostanay	4,28	>0,05	0,31	2,78	>0,05	0,37	2,43	**<0,05**	0,49
Kyzylorda	2,12	**<0,05**	0,56	0,66	**<0,01**	0,74	0,02	>0,05	0,19
Mangistau	1,38	>0,05	0,01	1,73	**<0,05**	0,69	-0,13	>0,05	0,07
South Kazakhstan	-5,80	**<0,01**	0,77	0,27	**<0,001**	0,91	0,01	>0,05	0,09
Pavlodar	6,33	>0,05	0,60	4,60	>0,05	0,39	2,08	**<0,05**	0,75
North Kazakhstan	-0,98	>0,05	0,19	2,45	**<0,05**	0,61	-2,78	**<0,01**	0,81
East Kazakhstan	2,02	>0,05	0,11	1,42	>0,05	0,33	3,64	**<0,001**	0,73
Almaty	3,02	**<0,05**	0,69	0,07	>0,05	0,43	3,12	**<0,05**	0,70
Astana	8,97	**<0,01**	0,78	6,99	**<0,01**	0,73	2,19	**<0,01**	0,76

Bold values mean statistical sagnificance (p-value<0.05).

The primary incidence of Childhood Autism among children across all regions has shown an upward trend from 2016 to 2022. Statistically significant increases were observed in the Akmola, Aktobe, West Kazakhstan, Zhambyl, Karaganda, Kyzylorda, Mangystau, South Kazakhstan, and North Kazakhstan regions, as well as in the city of Astana. The highest coefficient was recorded in Astana, where the primary incidence increased by an average of 6.99 per 100,000 children each year. The lowest significant increase was observed in the South Kazakhstan region, where the incidence rose by an average of 0.27 per 100,000 children annually.

The primary incidence of Atypical Autism among children from 2016 to 2022 showed a decline in the Mangystau and North Kazakhstan regions. The decrease in North Kazakhstan was statistically significant (b = -2.78; p-value < 0.01, CI=-4.15; -1.39). Conversely, statistically significant increases were observed in the Almaty, Atyrau, West Kazakhstan, Zhambyl, Karaganda, Kostanay, Pavlodar, North Kazakhstan, East Kazakhstan regions, and in the cities of Almaty and Astana. The highest coefficient was noted in the East Kazakhstan region, where the primary incidence of Atypical Autism increased by an average of 3.64 per 100,000 children annually. The smallest significant increase was recorded in the Almaty region, with an annual rise of 0.17 per 100,000 children.

### 3.2 Prevalence of neurodevelopmental disorders, including childhood and atypical autism, in the pediatric population from 2016 to 2022

The prevalence of childhood autism in Kazakhstan has seen a significant increase, rising from 14 per 100,000 children in 2016 to 59 per 100,000 children in 2022. A similar trend is observed with atypical autism, where the prevalence increased sixfold by 2022 compared to 2016.

The population of children with Neurodevelopmental disorders also shows a steady increase over the seven-year period, growing from 116.8 per 100,000 children in 2016 to 149.2 per 100,000 children in 2022.

**Figure 2:**
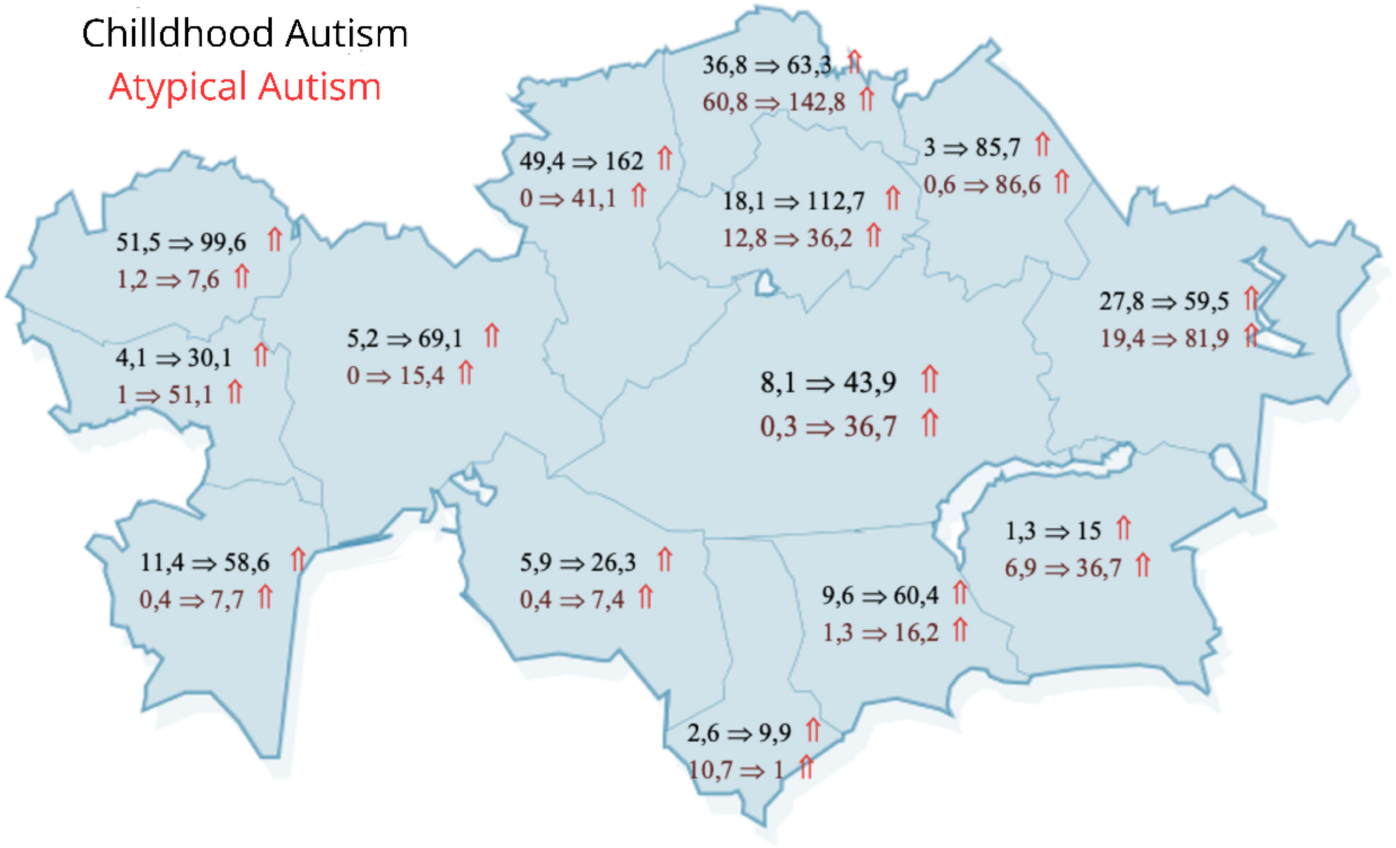
Changes in the prevalence of childhood and atypical autism per 100,000 children aged 0–17 years across regions of Kazakhstan in 2016 and 2022.

**Figure 2** illustrates the changes in the prevalence of childhood and atypical autism among children aged 0–17 years across all regions of Kazakhstan. The analysis revealed that in 2016, the highest prevalence of childhood autism was observed in the West Kazakhstan region, with a rate of 52.5 per 100,000 children. In contrast, the highest prevalence of atypical autism in 2016 was recorded in the North Kazakhstan region, at 60.8 per 100,000 children. By 2022, Akmola region emerged as the leader in the prevalence of childhood autism, with a rate of 112.7 per 100,000 children, while North Kazakhstan continued to lead in the prevalence of atypical autism among children, reporting a rate of 142.8 per 100,000. Overall, southern regions of Kazakhstan exhibit lower prevalence rates for both childhood and atypical autism compared to the northern regions.

The prevalence of childhood autism in cities of national significance varies significantly. In Astana, the prevalence of childhood autism has increased fourfold over seven years, reaching a rate of 263.7 per 100,000 children by 2022. In contrast, Almaty has experienced only a slight increase, with rates slightly exceeding 1 per 100,000 children in some years, and even falling below that threshold in others. In Shymkent, registration of children with childhood autism began in 2018, showing a notable increase to 57.1 per 100,000 children by 2022.

Conversely, the situation is different for atypical autism in these cities. Almaty has emerged as the leader in the prevalence of atypical autism, reporting a rate of 99.2 per 100,000 children in 2022. The city has shown a consistent increase in this diagnosis, rising from 9.3 per 100,000 children to 47.5 per 100,000 children by 2022. Meanwhile, Shymkent has seen a significant decline in the number of children diagnosed with atypical autism, with rates dropping from 32.8 per 100,000 children to 9 per 100,000 children in 2022.

## 4 Discussion

The present study offers a detailed analysis of the increasing incidence of childhood autism and atypical autism in Kazakhstan over a seven-year period. A notable observation is the contrast between the non-significant trend in the incidence of neurodevelopmental disorders, which showed a modest increase from 27.3 per 100,000 in 2016 to 34.1 per 100,000 in 2022, and the significant upward trends in childhood autism and atypical autism (p < 0.01). This divergence may indicate a plateau in the recognition of broader developmental disorders or, alternatively, a heightened focus on autism-specific diagnoses in recent years.

Nationally, childhood autism diagnoses have surged nearly fivefold, while atypical autism has increased fourfold. These trends align with international research suggesting that ASD are being diagnosed more frequently due to improved screening methods and greater awareness among healthcare professionals and the public (34–36). The statistically significant annual increase in both conditions further underscores the role of enhanced diagnostic practices in these rising trends. However, previous studies conducted in Kazakhstan have reported relatively low awareness of ASD among both healthcare workers and the general population (37, 38), suggesting that further improvements in awareness and training may still be needed.

Regional disparities in the incidence and prevalence of autism are also evident, with northern regions of Kazakhstan generally reporting higher rates of childhood and atypical autism compared to the southern regions. Several factors may contribute to these regional differences, including variations in healthcare infrastructure, socioeconomic conditions, and cultural attitudes toward autism. For example, the significant rise in the prevalence of childhood autism in Astana, reaching 263.7 per 100,000 children in 2022, underscores the role of urbanization and access to specialized healthcare services in the diagnosis of autism (39, 40).

Furthermore, environmental factors may play a role in these regional disparities. Previous studies have found that regions with a higher prevalence of ASD often coincide with areas characterized by industrial activity and air pollution (41–43). In Kazakhstan, assessments of air quality in major cities have shown that many exceed the permissible levels of hazardous pollutants, posing chronic health risks. Notably, cities such as Ust-Kamenogorsk, Shymkent, Aktobe, Almaty, and Petropavlovsk, which exhibit high levels of air pollution, also report elevated rates of childhood and/or atypical autism (44, 45). This suggests that environmental pollutants could be contributing factors to the regional differences in autism prevalence observed across Kazakhstan.

A meta-analysis of global autism prevalence found a cumulative rate of 72 per 10,000, with higher rates in North America (1.01%) compared to Europe (0.73%) and Asia (0.41%). Higher prevalence rates were linked to high-income countries with better diagnostic access and more rigorous study designs (23). An updated systematic review reported a global median prevalence of 65 per 10,000, slightly higher than previous estimates, with increasing rates in countries like the USA, South Korea, and Australia (16). A systematic review of 74 studies covering over 30 million people revealed prevalence rates ranging from 0.02% in China to 3.66% in Sweden, with regional rates of 0.4% in Asia, 1% in the Americas, 0.5% in Europe, 1% in Africa, and 1.7% in Australia (27). While autism prevalence in high-income countries ranges from 0.9% to 1.5%, global estimates remain lower at 0.6%, likely reflecting underdiagnosis due to limited services (46). Recent analysis of global disease burden data (1990–2019) showed stable prevalence rates in North Africa and the Middle East (47). Although autism is increasingly recognized globally, differences in registered prevalence highlight gaps in awareness, diagnosis, and research between high- and low-income countries. High-income countries consistently report higher prevalence due to better surveillance systems, trained specialists, and public awareness (48) In contrast, low- and middle-income countries report lower and more variable prevalence rates, often due to inconsistent diagnostic capacities (49, 50). Low-income countries report the lowest rates, often below 0.2%, due to underdiagnosis, limited access to services, and lack of culturally adapted screening tools (51, 52). In Kazakhstan, the prevalence of childhood and atypical autism is reported as 96.3 per 100,000, or 0.09%, which is significantly lower than even low-income countries, but likely does not reflect true prevalence. A study conducted in Central Kazakhstan on autism risk assessment among children undergoing routine developmental screening showed a high-risk prevalence of 27.4% (53). This discrepancy may be attributed to factors such as:

- Low awareness among healthcare professionals, as many rely on subjective assessments like behavior observation and outdated diagnostic criteria (e.g., ICD-10) with limited use of standardized tools like ADOS due to lack of training. Cultural stigma around autism, as well as misconceptions linking it to schizophrenia or poor parenting, further contribute to misdiagnosis and underdiagnosis in the country (54).
- Low public awareness of autism, leading parents to seek help from unqualified specialists when concerns arise (55).
- Underfunding of research, especially in Central Asia, including Kazakhstan (56).
- In Kazakhstan, there is significant stigmatization associated with seeking psychiatric care, particularly for children, which often deters families from consulting child psychiatrists. As a result, many children are assessed by non-specialists, leading to misdiagnoses and underreporting (57).
- Diagnosis replacement: In Kazakhstan, autism diagnoses are only assigned to children under 18, meaning the official statistics cover only minors. Upon reaching adulthood, individuals diagnosed with autism are often reassigned to schizophrenia, especially in contexts like military service exemptions (58).

A growing body of research shows that many parents recognize signs of autism long before an official diagnosis, often due to their need for access to services (59). However, parents tend to describe their concerns using general terms that may not align with clinical diagnostic criteria yet still reflect critical observations. Early concerns are often related to issues such as sleep disturbances, activity levels, and emotional regulation rather than typical ASD symptoms. It is crucial to consider all parental concerns, as they may signal early atypical development. Focusing solely on stereotypical signs can delay diagnosis, making it essential to incorporate gender, social expectations, and socio-economic factors in interpretation. Clinicians must be trained to accurately interpret parental observations to facilitate early detection and support (60, 61).

Recent research conducted in Kazakhstan highlights a significant lack of awareness regarding ASD, its prevalence, and available support services, underscoring the urgent need for large-scale educational initiatives aimed at improving early recognition and reducing stigma (62, 63). Future initiatives should focus on targeted support for first-time parents who may lack developmental benchmarks, as well as culturally sensitive training for healthcare providers to ensure effective communication with families from diverse backgrounds. Strengthening partnerships between families and healthcare providers can build trust, promote proactive engagement, and ultimately improve outcomes for children with ASD. Social marketing strategies, behavior change theory, and audience research can play a key role in facilitating early detection and intervention for autism and other developmental disorders (64).

One limitation of this study is its reliance on secondary data from mental health centers, which may not capture the full spectrum of ASD diagnoses due to potential underreporting or variations in diagnostic practices across regions. Additionally, the study focuses on data from Kazakhstan, which may limit the generalizability of the findings to other countries with different healthcare systems, socio-cultural factors, and diagnostic criteria. The retrospective nature of the analysis may also hinder the ability to explore causal relationships between environmental factors, healthcare access, and the rising prevalence of ASD.

## Data Availability

The original contributions presented in the study are included in the article/supplementary material. Further inquiries can be directed to the corresponding author.

## References

[1] Brinkman S Stanley F. Public health aspects of child well-being. Handb Child Well-Being Theor Methods Policies Glob Perspect. (2014) 1:317–50. doi: 10.1007/978-90-481-9063-8_16

[2] United Nations Children's Fund. Early childhood development: UNICEF vision for every child. Early Child Dev UNICEF Vis Every Child. (2023). doi: 10.18356/9789213585429

[3] Scott JG Mihalopoulos C Erskine HE Roberts J Rahman A. Childhood mental and developmental disorders. Dis control priorities. Ment Neurol Subst Use Disord. (2016) Volume 4):145–61. doi: 10.1596/978-1-4648-0426-7_CH8 27227241

[4] Olusanya BO Smythe T Ogbo FA Nair MKC Scher M Davis AC. Global prevalence of developmental disabilities in children and adolescents: A systematic umbrella review. Front Public Heal. (2023) 11:1122009/FULL. doi: 10.3389/FPUBH.2023.1122009/FULL PMC9987263 36891340

[5] UNICEF. Organizacion Mundial de la Salud. In: Global report on children with developmental disabilities. Geneva: World Health Organization and the United Nations Children’s Fund (2023).

[6] Zafeiriou DI Ververi A Vargiami E. Childhood autism and associated comorbidities. Brain Dev. (2007) 29:257–72. doi: 10.1016/j.braindev.2006.09.003 17084999

[7] Jagadapillai R. Atypical autism: causes, diagnosis and support. Medicina (Kaunas). (2024) 60:1163. doi: 10.3390/medicina60071163 39064592 PMC11278543

[8] Kanai C Koyama T Kato S Miyamoto Y Osada H Kurita H. Comparison of high-functioning atypical autism and childhood autism by Childhood Autism Rating Scale–Tokyo version. Psychiatry Clin Neurosci. (2004) 58:217–21. doi: 10.1111/j.1440-1819.2003.01220.x 15009830

[9] Hosseini SA Molla M. Asperger syndrome. In: StatPearls [Internet]. StatPearls Publishing, Treasure Island (FL (2025). Available at: https://www.ncbi.nlm.nih.gov/books/NBK557548/. 32491480

[10] American Psychiatric Association. Diagnostic and statistical manual of mental disorders (DSM-5-TR). Washington: American Psychiatric Association. (2022).

[11] Oberman LM Kaufmann WE. Autism spectrum disorder versus autism spectrum disorders: terminology, concepts, and clinical practice. Front Psychiatry. (2020) 11:484. doi: 10.3389/fpsyt.2020.00484 32636765 PMC7317665

[12] ICD-11. International classification of diseases 11th revision . Available online at: https://icd.who.int/en/ (Accessed April 10, 2025).

[13] Greaves-Lord K Skuse D Mandy W. Innovations of the ICD-11 in the field of autism spectrum disorder: A psychological approach. Clin Psychol Eur. (2022) 4:e10005. doi: 10.32872/cpe.10005 36760320 PMC9881114

[14] Hodges H Fealko C Soares N. Autism spectrum disorder: definition, epidemiology, causes, and clinical evaluation. Transl Pediatr. (2020) 9:S55. doi: 10.21037/TP.2019.09.09 32206584 PMC7082249

[15] Yeargin-Allsopp M. Past and Future perspectives in autism epidemiology. Mol Psychiatry. (2002) 7(2):S9–S11. doi: 10.1038/sj.mp.4001164 12142933

[16] Zeidan J Fombonne E Scorah J Ibrahim A Durkin MS Saxena S. Global prevalence of autism: A systematic review update. Autism Res. (2022) 15:778–90. doi: 10.1002/AUR.2696 PMC9310578 35238171

[17] Al-Beltagi M. Autism medical comorbidities. World J Clin Pediatr. (2021) 10:15. doi: 10.5409/WJCP.V10.I3.15 33972922 PMC8085719

[18] KhaChadourian V Mahjani B Sandin S Kolevzon A Buxbaum JD Reichenberg A. Comorbidities in autism spectrum disorder and their etiologies. Transl Psychiatry. (2023) 131:2023. doi: 10.1038/s41398-023-02374-w PMC9958310 36841830

[19] Lavelle TA Weinstein MC Newhouse JP Munir K Kuhlthau KA Prosser LA. Economic burden of childhood autism spectrum disorders. Pediatrics. (2014) 133:e520–9. doi: 10.1542/PEDS.2013-0763 PMC7034397 24515505

[20] Rogge N Janssen J. The economic costs of autism spectrum disorder: A literature review. J Autism Dev Disord. (2019) 49:2873–900. doi: 10.1007/S10803-019-04014-Z/METRICS 30976961

[21] Taneja A Sharma S Bhatt N Bhutani M. Economic burden of autism and autism-related spectrum disorders (Asd) in EU5 countries. Value Heal. (2017) 20:A712. doi: 10.1016/j.jval.2017.08.1885

[22] Khaitova AI Goncharova NA Makarova EN. Financial and economic prospects for the socialization of children with autism spectrum disorders. Vestn Astrakhan State Tech Univ Ser Econ. (2023) 2023:128–34. doi: 10.24143/2073-5537-2023-3-128-134

[23] Talantseva OI Romanova RS Shurdova EM Dolgorukova TA Sologub PS Titova OS. The global prevalence of autism spectrum disorder: A three-level meta-analysis. Front Psychiatry. (2023) 14:1071181/BIBTEX. doi: 10.3389/FPSYT.2023.1071181/BIBTEX 36846240 PMC9947250

[24] Chinawa JM Manyike PC Aniwada EC Chinawa AT Obu HA Odetunde OI. Prevalence and socioeconomic correlates of autism among children attending primary and secondary schools in south east Nigeria. Afr Health Sci. (2016) 16:936. doi: 10.4314/AHS.V16I4.8 28479884 PMC5398438

[25] Kakooza-Mwesige A Ssebyala K Karamagi C Kiguli S Smith K Anderson MC. Adaptation of the ‘ten questions’ to screen for autism and other neurodevelopmental disorders in Uganda. Autism. (2014) 18:447–57. doi: 10.1177/1362361313475848 23536263

[26] Nasledov AD Miroshnikov S Zashchirinskaia OV Tkacheva L Kompanets NN. Autism Scale application for identifying the risk of mental development disorders among children ages 3 and 4. Sib Psikhologicheskiy Zhurnal. (2022) 83):164–83. doi: 10.17223/17267080/83/9

[27] Salari N Rasoulpoor S Rasoulpoor S Shohaimi S Jafarpour S Abdoli N. The global prevalence of autism spectrum disorder: a comprehensive systematic review and meta-analysis. Ital J Pediatr. (2022) 481:2022. doi: 10.1186/S13052-022-01310-W PMC9270782 35804408

[28] Fefelov V. V SSSR invalidov net! (There are no invalids in the USSR)!. London: Overseas Publications Interchange Ltd (1986).

[29] Tomini SM Vanore M Yousefzadesh S Gassman F. Situation analysis of children with disabilities for the development of an inclusive society in the republic of Kazakhstan, - astana. Republic of Kazakhstan, Astana: United Nations Children’s Fund (UNICEF) (2014) 108.

[30] Dunn SP Dunn E. Everyday Life of people with disabilities in the USSR. In: McCagg WO Siegelbaum L, editors. People with disabilities in the soviet union: past and present, theory and practice. University of Pittsburgh Press, Pittsburgh (1989). p. 199–234.

[31] Poloziuk O. Problems of socio-legal protection of disabled persons with spinal cord injuries in Ukraine, in: Paper presented at the Sixth Congress of the International Association of Ukrainian Studies, Donetsk, Ukraine, June 29-July 1, 2005. (2005).

[32] General disorders of psychological (mental) development. In: Autism spectrum disorders. Clinical Protocol. Ministry of Health Republic of Kazakhstan, Astan (2021). Available online at: https://nrchd.kz/ru/2017-03-12-10-51-13/klinicheskie-protokoly.

[33] Minister of Health Republic of Kazakhstan. Order of the minister of health of the republic of Kazakhstan dated november 30, 2020. In: Registered in the Ministry of Justice of the Republic of Kazakhstan on December 2, 2020 No. 21712 “On approval of the standard for the organization of the provision of medical and social assistance in the field of mental health to the population of the Republic of Kazakhstan. Ministry of Health of Republic of Kazakhstan, Astana. p. 224.

[34] Wang Y. Research on the culture and awareness of autism. Trans Soc Sci Educ Humanit Res. (2024) 6:303–11. doi: 10.62051/ME5AD084

[35] Hidiroglu S Luleci NE Karavus M Tanriover O Bayar ES. The awareness of childhood autism among residents of neuropsychiatric and other disciplines of a research and training hospital in Istanbul. Journal of Pakistan Medical Association, Karachi: Turkey-Web of Science Core Collection (2024). Available online at: https://www.webofscience.com/wos/woscc/full-record/WOS:000432456900017 (Accessed August 31, 2024). 29479101

[36] Kazimirova OV Khaidargalieva LS Zhaparkul BD Mukhametzhanova RA Aitzhanova GA Yugay MN. Screening diagnostics methods in family doctor practice. Med ecology. (2021) 2):6–18.

[37] Pather S Somerton M Jaxybayeva A Stolyarova V Khanin S. Diagnosing children with intellectual impairment and autism in Kazakhstan. Nazarbayev University, Astana.

[38] Kurmanalina S Samambayeva A Akhtayeva N Kozhageldiyeva L Kosherbayeva L. Awareness of autism spectrum disorder among population of Kazakhstan. J Autism Dev Disord. (2024). doi: 10.1007/S10803-024-06350-1 38656465

[39] Shenouda J Barrett E Davidow AL Halperin W Silenzio VMB Zahorodny W. Prevalence of autism spectrum disorder in a large, diverse metropolitan area: Variation by sociodemographic factors. Autism Res. (2022) 15:146–55. doi: 10.1002/AUR.2628 PMC8755586 34672116

[40] Divan G Bhavnani S Leadbitter K Ellis C Dasgupta J Abubakar A. Annual Research Review: Achieving universal health coverage for young children with autism spectrum disorder in low- and middle-income countries: a review of reviews. J Child Psychol Psychiatry. (2021) 62:514–35. doi: 10.1111/JCPP.13404 33905120

[41] Moschetti A Giangreco M Ronfani L Cervellera S Ruffilli MP Nume C. An ecological study shows increased prevalence of autism spectrum disorder in children living in a heavily polluted area. Sci Rep. (2024) 141:2024. doi: 10.1038/s41598-024-67980-0 PMC11282186 39060326

[42] Weisskopf MG Kioumourtzoglou MA Roberts AL. Air pollution and autism spectrum disorders: causal or confounded? Curr Environ Heal Rep. (2015) 2:430–9. doi: 10.1007/S40572-015-0073-9/FIGURES/3 PMC4737505 26399256

[43] Dutheil F Comptour A Morlon R Mermillod M Pereira B Baker JS. Autism spectrum disorder and air pollution: A systematic review and meta-analysis. Environ Pollut. (2021) 278:116856. doi: 10.1016/J.ENVPOL.2021.116856 33714060

[44] Kenessary D Kenessary A Adilgireiuly Z Akzholova N Erzhanova A Dosmukhametov A. Air pollution in Kazakhstan and its health risk assessment. A Ann Glob Heal. (2019) 85:133. doi: 10.5334/AOGH.2535 PMC6838766 31750082

[45] Kerimray A Assanov D Kenessov B Karaca F. Trends and health impacts of major urban air pollutants in Kazakhstan. J Air Waste Manage Assoc. (2020) 70:1148–64. doi: 10.1080/10962247.2020.1813837 32841107

[46] Samadi SA. Overview of services for autism spectrum disorders (ASD) in low- and middle-income countries (LMICs) and among immigrants and minority groups in high-income countries (HICs). Brain Sci. (2022) 12:1682. doi: 10.3390/brainsci12121682 36552142 PMC9775866

[47] Ebrahimi Meimand S Amiri Z Shobeiri P Malekpour MR Saeedi Moghaddam S Ghanbari A. Burden of autism spectrum disorders in North Africa and Middle East from 1990 to 2019: A systematic analysis for the Global Burden of Disease Study 2019. Brain Behav. (2023) 13:e3067. doi: 10.1002/brb3.3067 37350023 PMC10338812

[48] Christensen DL Maenner MJ Bilder D Constantino JN Daniels J Durkin MS. Prevalence and characteristics of autism spectrum disorder among children aged 4 years - early autism and developmental disabilities monitoring network, seven sites, United States, 2010, 2012, and 2014. MMWR Surveill Summ. (2019) 68:1–19. doi: 10.15585/mmwr.ss6802a1 PMC6476327 30973853

[49] Hong M Lee SM Park S Yoon SJ Kim YE Oh IH. Prevalence and economic burden of autism spectrum disorder in South Korea using national health insurance data from 2008 to 2015. J Autism Dev Disord. (2020) 50:333–9. doi: 10.1007/s10803-019-04255-y 31630294

[50] Paula CS Ribeiro SH Fombonne E Mercadante MT. Brief report: prevalence of pervasive developmental disorder in Brazil: a pilot study. J Autism Dev Disord. (2011) 41:1738–42. doi: 10.1007/s10803-011-1200-6 21337063

[51] Chinawa JM Manyike PC Aniwada EC Chinawa AT Obu HA Odetunde OI. Prevalence and socioeconomic correlates of autism among children attending primary and secondary schools in south east Nigeria. Afri Health Sci. (2016) 16:936942. doi: 10.4314/ahs.v16i4.8 PMC5398438 28479884

[52] Samadi SA McConkey R. Autism in developing countries: lessons from Iran. Autism Res Treat. (2011) 2011:145359. doi: 10.1155/2011/145359 22937242 PMC3420542

[53] Nukeshtayeva K Omarkulov B Lyubchenko M Delellis N Zhamantayev O Dauletkaliyeva Z. Prevalence of Autism risk among children undergoing regular Psychophysical Development Screening in Kazakhstan. Clin Epidemiol Glob Heal. (2024) 28:145359. doi: 10.1016/j.cegh.2024.101703

[54] Somerton M Stolyarova V Khanin S. Autism and the knowledge and beliefs of specialists in Kazakhstan. J Autism Dev Disord. (2022) 52:1156–68. doi: 10.1007/s10803-021-05021-9 33890202

[55] Kurmanalina S Samambayeva A Akhtayeva N Kozhageldiyeva L Kosherbayeva L. Awareness of autism spectrum disorder among population of Kazakhstan. J Autism Dev Disord. (2024). doi: 10.1007/s10803-024-06350-1 38656465

[56] Zakirova-Engstrand R Yakubova G. A scoping review of autism research conducted in Central Asia: Knowledge gaps and research priorities. Autism. (2023) 28:342–54. doi: 10.1177/13623613231170553 PMC10851649 37161788

[57] Nukeshtayeva K Omarkulov B Lyubchenko M Delellis N Mussina A Kayupova G. Untangling the path: challenges in autism diagnosis for Kazakhstani families. Astana Med J. (2024) 3:19–26. doi: 10.54500/2790-1203-2024-3-122-19-26

[58] Pather S Somerton M Jaxybayeva A Stolyarova V Khanin S. Diagnosing children with intellectual impairment and autism in Kazakhstan. In: Faculty grant project, graduate school of education, nazabayev university. Project report. Nazarbayev University, Astana.

[59] Gentles SJ Nicholas DB Jack SM McKibbon KA Szatmari P. Coming to understand the child has autism: A process illustrating parents’ evolving readiness for engaging in care. Autism. (2019) 24:470–83. doi: 10.1177/1362361319874647 PMC6985991 31508991

[60] Guinchat V Chamak B Bonniau B Bodeau N Perisse D Cohen D. Very early signs of autism reported by parents include many concerns not specific to autism criteria. Res Autism Spectr Disord. (2012) 6:589–601. doi: 10.1016/j.rasd.2011.10.005

[61] Yu LM. Experience of implementation of integrative system family approach to programs of continuous professional development of mental profile specialists. Med ecology. (2018) 2):131–4.

[62] Kosherbayeva L Kurmanalina S Akhtaeva N Samambayeva A Altynbekov K. Understanding autism spectrum disorders among the population of Kazakhstan//Nauka i Zdravookhranenie. Sci Healthcare. (2023) 25:133–9. doi: 10.34689/SH.2023.25.4.016

[63] Nukeshtayeva K Lyubchenko M Omarkulov B Delellis N. Modified checklist for autism in toddlers, revised, with follow-up application in Central Kazakhstan. J Clin Med Kaz. (2022) 19:36–41. doi: 10.23950/jcmk/11574

[64] Daniel KL Prue C Taylor MK Thomas J Scales M. ‘Learn the signs. Act early’: a campaign to help every child reach his or her full potential. Public Health. (2009) 123 Suppl 1:e11–6. doi: 10.1016/j.puhe.2009.06.002 19767041

